# Wheat grain proteins: Past, present, and future

**DOI:** 10.1002/cche.10585

**Published:** 2022-07-30

**Authors:** Peter Shewry

**Affiliations:** ^1^ Rothamsted Research Harpenden, Hertfordshire UK

**Keywords:** amylase/trypsin inhibitor (ATI), gluten, proteins, puroindolines, wheat

## Abstract

**Background and Objectives:**

Research on wheat grain proteins is reviewed, including achievements over the past century and priorities for future research. The focus is on three groups of proteins that have major impacts on wheat quality and utilization: the gluten proteins which determine dough viscoelasticity but also trigger celiac disease in susceptible individuals, the puroindolines which are major determinants of grain texture and the amylase/trypsin inhibitors which are food and respiratory allergens and are implicated in triggering celiac disease and nonceliac wheat sensitivity.

**Findings:**

Although earlier work focused on protein structure and properties, the development of genomics and high‐sensitivity proteomics has resulted in the availability of a vast amount of information on the amino acid sequences of individual wheat proteins, including allelic variants of gluten proteins which are associated with good processing quality and of puroindolines, which are associated with a hard or soft grain texture, and on protein expression and polymorphism.

**Conclusions:**

However, our ability to exploit this knowledge is limited by a lack of detailed understanding of the structure:function relationships of wheat proteins. In particular, we need to understand how the three‐dimensional structures of the individual proteins determine their interactions with other grain components (to determine functional properties) and with the immune systems of susceptible consumers (to trigger adverse responses), how these interactions are affected by allelic variation, and how they can be manipulated.

**Significance and Novelty:**

The article, therefore, identifies priorities for future research which should enable the adoption of a more rational approach to improving the quality of wheat grain proteins.

## 1 INTRODUCTION

Although Cereal Chemistry publishes papers on a range of grain crops and topics, there is no doubt that wheat has been the dominant crop in terms of numbers of publications, and wheat proteins the dominant topic. The number of papers published over the past century has been vast: a simple online search using the Web of Knowledge database showed that 3307 papers were published on wheat (as a “topic”) in Cereal Chemistry between 1945 and March 2022, of which 1678 papers included the words “wheat protein” or “gluten.” Based on these numbers alone (and ignoring publications before 1945 and in other journals) a cynic could ask how much more do we really need to know about wheat grain proteins?

The answer to this is that our knowledge is still far from complete. This is because although we know a lot about some aspects of wheat grain proteins, we know much less about other aspects, such as their three‐dimensional structures and structure:function relationships.

In this article, I will celebrate the massive contributions that Cereal Chemistry has made to our knowledge of wheat grain proteins and highlight the contributions of my colleague Craig Morris (1957–2021). I will initially discuss the development of wheat protein chemistry over the past century and then focus on three groups of proteins that have significant impacts on processing quality and health: gluten proteins, puroindolines, and α‐amylase/trypsin inhibitors (ATIs).

## 2 HISTORICAL OVERVIEW

The long history of wheat protein research is well documented, from the first description of gluten published in 1745 by Jacopo Beccari (Professor of Chemistry at the University of Bologna) in his article “De frumento” (“concerning corn or grain”) (Bailey, 1941). Further studies were reported in the 18th and early 19th centuries, a notable advance being made by Taddei (1819) who separated gluten into fractions that were soluble (gliadins) or insoluble (zymon, later called glutenin) in alcohol. However, the first detailed systematic studies were reported by Thomas Burr Osborne (1859–1929), one of the founding fathers of protein chemistry, who published some 250 papers on plant proteins, including studies of seed proteins from 32 species, over the period from 1886 to 1928. His studies of wheat are recorded in research papers and in his monograph *The Vegetable Proteins* (Osborne, 1924). Osborne concluded that the proteins present in plant tissues comprised four major types which have since become known as “Osborne fractions”: albumins, globulins, prolamins, and glutelins.

Further advances came in the second half of the 20th century with the development of methods to separate and purify individual proteins: electrophoresis in starch gel (Jones et al., 1959), sodium dodecylsulphate polyacrylamide gel electrophoresis (SDS‐PAGE) (Bietz & Wall, 1972), two‐dimensional electrophoresis (Wrigley & Shepherd, 1973), the use of chaotropic agents (Meredith & Wren, 1966), and high‐performance liquid chromatography (HPLC) (Bietz, 1983). At the same time, gluten protein fractions were subjected to biophysical analysis to determine aspects of their structures and properties and partial sequences were determined using automated Edman degradation (notably at the Northern and Western Regional Research Centres of the USDA at Peoria and Albany, respectively) (see Shewry & Hamer (2014) for a brief overview).

However, the most dramatic advances came with the development, starting in the 1980s, of “omics” technologies, notably the rapid automated sequencing of complementary DNA (cDNA) and genomic DNA and high‐sensitivity mass spectrometry‐based proteomics. This combination of approaches has led to a massive explosion in our knowledge of the sequences of wheat proteins, including variation within and between the multigene families encoding wheat gluten proteins. For example, S. Bromilow, Gethings, Buckley, et al. (2017) assembled a curated database of 630 gluten protein sequences from over 24,000 gluten‐related sequences in the UniProt database. However, this increase in information has not been reflected in a similar increase in our understanding of protein structure and function.

## 3 GLUTEN PROTEINS AND PROCESSING QUALITY

### 3.1 What do we know?

As discussed above, the main focus of wheat protein research over the past century has been on the gluten protein fraction, with the main purpose being to understand the basis for the unique biophysical properties (viscosity combined with extensibility and elasticity) of wheat dough in order to underpin quality improvement by crop genetic improvement or innovative processing. Gluten is a complex mixture, with between 50 and 100 individual proteins being separated by two‐dimensional electrophoresis. These are classically divided into two groups, the monomeric gliadins (classically defined as prolamins), and polymeric glutenins (glutelins), and into protein types within these groups: the high molecular and low‐molecular‐weight subunits of glutenin (HMW‐GS and LMW‐GS, respectively) and the α‐type gliadins, γ‐gliadins, and ω‐gliadins. We now have essentially full sets of amino acid sequences of these proteins for individual wheat genotypes (see e.g., S. N. L. Bromilow, Gethings, Langridge, et al. (2017)).

When wheat flour is mixed with water to form dough the gluten proteins form a continuous three‐dimensional network which is stabilized by both covalent and noncovalent forces. The glutenin polymers are stabilized by covalent interchain disulfide bonds, some of which have been identified by mass spectrometry (see, e.g., Schmid et al., 2017). The glutenin polymers vary in molecular mass from oligomers of mass 100,000 to 150,000 to polymers with masses up to at least 1–2 million with the HMW‐GS being concentrated in high molecular mass polymers (Shewry & Lafiandra, 2022). Furthermore, larger “aggregates” also occur which comprise glutenin polymers and gliadins (Morel et al., 2020). These are stabilized by noncovalent forces, with hydrogen bonds being the most important (although hydrophobic and electrostatic interactions may also contribute).

Glutenin polymers can also be prepared as a hydrated gel after the extraction of flour with 1.5% SDS. This fraction has been termed “glutenin macropolymer” (GMP) and the amount shown to correlate with breadmaking quality (Weegels et al., 1996). GMP clearly has a much higher mass than the “aggregates” prepared by Morel et al. (2020) and the relationships between the structure of GMP, the glutenin polymers present in the dry flour and the hydrated network in dough remain to be established.

In view of this complexity, it is not surprising that the precise relationships between the structure and properties of gluten are incompletely understood. Viscosity may result from noncovalent bonding between monomers and polymers, principally hydrogen bonding between glutamine residues which account for between about 20% and 50% of the total amino acids of individual gluten proteins. These glutamine residues are regularly arranged in the protein repetitive domains which may allow the formation of “glutamine zips,” as demonstrated for protein deposits in neurodegenerative diseases of humans (Perutz et al., 1994). Similarly, dough extensibility may result from slippage between the noncovalently bound gliadins and glutenin polymers when force is applied.

The molecular basis for elasticity is less well understood but is almost certainly more complex. The importance of interchain disulfide bonds is indisputable as elasticity is lost if these are reduced. However, this does not prove that they contribute to the elastic mechanism, as opposed to stabilizing the elastic polymers, and two other mechanisms have been proposed.

The first is that HMW‐GS molecules are intrinsically elastic due to the formation of a loose “β‐spiral” super‐secondary structure which is based on regularly repeated β‐reverse turns (Tatham et al., 1985). The intrinsic elasticity of these structures has been demonstrated by analysis of model peptides based on the HMW‐GS repetitive domains, by deformation of HMW‐GS after cross‐linking (Tatham et al., 2001), and by stretching individual HMW‐GS molecules using an atomic force microscope (Haward et al., 2011).

**Figure 1:**
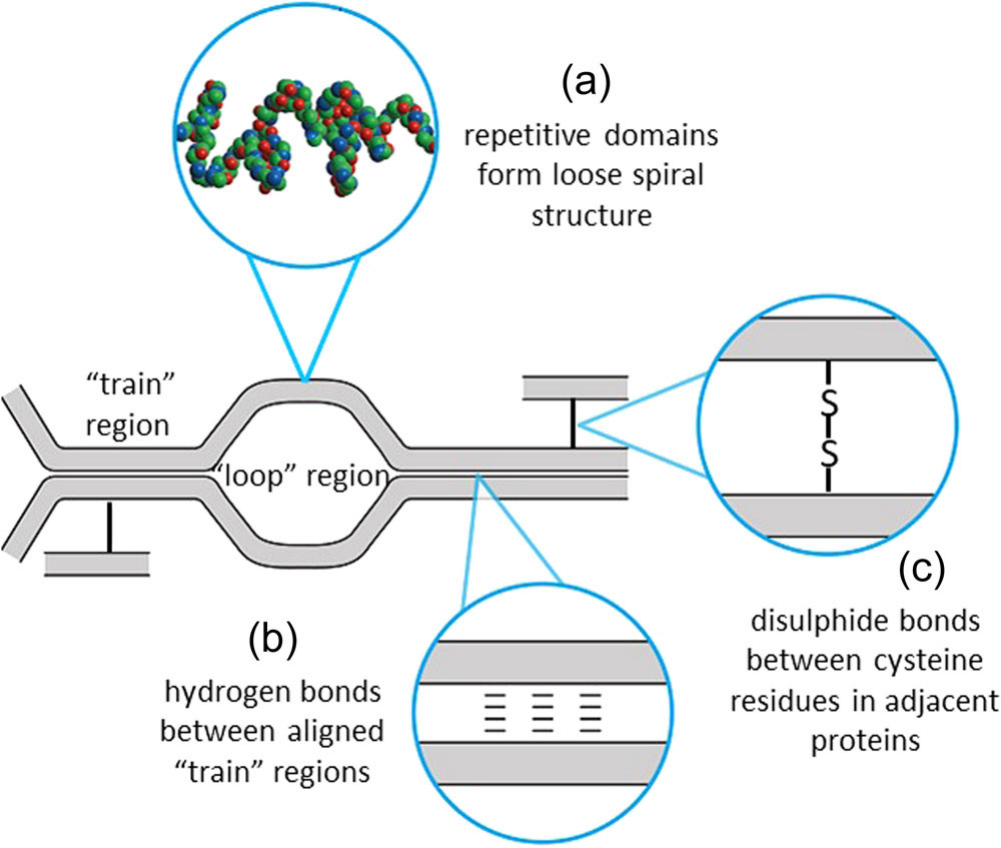
Schematic diagram summarising features of HMW‐GS polymers which may contribute to the elastic mechanism of gluten. (a) the loose β‐spiral structure formed by the repetitive sequences present in HMW‐GS may be intrinsically elastic, acting as a “molecular spring” (molecular model taken from Parchment et al., 2001); (b) the formation of “loop and train regions,” the latter stabilized by “glutamine zips” (arrays of hydrogen bonds between glutamine residues) may result in an equilibrium state which is disrupted when force is applied, resulting in reformation of the equilibrium state when the force is relaxed; (c) interchain disulfide bonds stabilize the polymer structure and may become stretched when force is applied, resulting in recoil when the force is relaxed. [Color figure can be viewed at wileyonlinelibrary.com]

However, Belton has suggested that hydrogen bonding between adjacent proteins also contributes to elasticity (Belton, 1995, 2005). He suggested that dry gluten is disordered but that regular hydrogen‐bonded structures are formed on hydration by orientation of the β‐turns in adjacent β‐spirals to form structures resembling “interchain” β‐sheet. Further hydration results in the replacement of some of the interchain hydrogen bonds with water, resulting in an equilibrium between aligned regions (trains) and loop regions. Mechanical deformation of this structure will initially extend the loops but eventually also separate the train regions, resulting in restoration of the equilibrium between loops and trains when the force is released. Finally, disulfide bonds may also contribute, as mechanical stress will result in deformation and a return to the undeformed structure on release. Hence, elasticity will be affected by differences in the sequences of the glutamine‐rich repetitive domains of the gluten proteins as well as the distributions of cysteine residues and their ability to form interchain disulfide bonds. These three mechanisms are depicted in Figure 1.

Readers are referred to two recent review articles for more detailed accounts of the structure and properties of wheat glutenin polymers (Shewry & Lafiandra, 2022; Lafiandra & Shewry, 2022). However, the aim here is to highlight some of the outstanding gaps in our knowledge.

### 3.2 What do we need to know?

It is possible to identify a number of knowledge gaps that are limiting our ability to improve the processing quality of wheat grain.

Three of these are briefly discussed.

#### 3.2.1 What are the precise structures of glutenin polymers and how are they determined by the structures and properties of the individual subunits?

Correlations between the allelic forms of HMW‐GS and breadmaking quality were established over 30 years ago (Payne, 1987) and similar relationships between some LMW‐GS and pasta‐making quality have since been reported (Oak & Dexter, 2006). We also know that the presence of some “good quality” HMW‐GS is associated with increased proportions of high‐molecular‐mass glutenin polymers (Lemelin et al., 2005; Naeem & MacRitchie, 2005). Two of the HMW‐GS associated with good quality (1Dx5, 1Bx7) have higher numbers of cysteine residues compared with the allelic poor‐quality subunits (1Dx2 and 1Bx20, respectively) which may result in more highly cross‐linked, and hence more elastic, polymers. In other cases, the good quality associated with individual subunits may result from quantitative effects of gene expression levels on total HMW subunit amount, for example, the higher quality resulting from the presence of a 1Ax subunit (1Ax1 or 1Ax2*) compared with the null allele, or the overexpression of subunit 1Bx7^OE^ (which results from a gene duplication) compared with the normal allele (reviewed by Lafiandra & Shewry, 2022). However, these mechanisms do not provide explanations for all reported associations of HMW subunits with grain quality and it is probable that more subtle differences in the amino acid sequences of HMW‐GS also contribute, for example, by affecting the formation of noncovalent hydrogen bonds (and hence “train” regions in hydrated gluten as discussed above). A greater understanding of these differences should allow the structures and properties of the polymers to be “fine‐tuned” by gene editing rather than the gross changes resulting from transgenesis and mutagenesis.

#### 3.2.2 What are the relationships between the structures of the glutenin polymers present in the grain, dough, and isolated fractions?

The gluten proteins are deposited in the starchy endosperm cells of the mature grains as discrete protein bodies. These protein bodies have two origins, with the HMW‐GS being concentrated in bodies formed within the lumen of the endoplasmic reticulum while gliadins (and probably also LMW‐GS) are concentrated in protein bodies of vacuolar origin (Tosi, 2012). The protein bodies fuse during the later stages of grain development, with the gluten proteins within a single endosperm cell forming a continuous matrix. The growth and rearrangement of glutenin polymers, including the formation of disulfide bonds between individual polymers, may occur in the protein bodies and in the protein matrix during grain maturation, perhaps catalyzed by low‐molecular‐weight redox reagents (Branlard et al., 2020).

The addition of water and mixing brings the gluten matrices in the individual cells together to form a network, with the input of mechanical energy and an increase in gluten protein hydration from about 15%–50% dry wt. Further mechanical input and washing are used to prepare gluten from the dough, either commercially or in the laboratory, while various treatments (such as stirring, sonication, and the use of organic solvents, detergents, and chaotropic agents) may be used to prepare “glutenin polymers” from a range of materials: grain, flour, dough, and gluten. All of these processes may have effects on the covalent structures and noncovalent interactions of glutenin polymers and we need to understand these if we wish to use rational design to improve quality.

#### 3.2.3 What are the interactions of gluten with other grain components?

The predominant role of the gluten proteins in determining dough properties and breadmaking quality has resulted in the comparative neglect of other grain components by researchers. However, it is clear that other grain components also affect functionality, and that some may interact with the gluten network to modulate its properties.

Vital gluten isolated by commercial starch/gluten fractionation is known to contain other proteins, which may be bound or entrapped. The presence of these proteins may reflect the interactions of the proteins in the dough. For example, Kobrehel et al. (1988) reported that forms of ATIs were noncovalently associated with glutenin (which they called durum‐wheat sulfur‐rich glutenin, DSG) and suggested that they affected the cooking quality of pasta (Kobrehel & Alari, 1989). Similarly, intrinsic lipids of wheat flour bind to gluten with effects on functionality (reviewed by Pareyt et al., 2011).

It is notable that most of the studies discussed above were carried out over 10 years ago and there has been relatively little fundamental work on gluten structure since then. This contrasts with advances in wheat biotechnology, which allow gluten protein composition to be manipulated using mutagenesis, transgenesis, or gene editing (reviewed by Shewry et al., 2022). A greater understanding of the structure:functionality relationships of gluten would clearly be of benefit in defining the targets for these studies as well as interpreting the impacts.

### 3.3 How can we answer these questions?

Studies of gluten structure and functionality are exceptionally challenging as hypotheses can only be fully explored in the context of the whole system. However, it is possible to study some individual factors using model systems. For example, Wellner et al. (2006) explored the relationships between the sequences, hydrogen bonding, and solubility of sequences present in HMW subunit repetitive domains using recombinant peptides expressed in *E. coli*. This approach could be used to explore other aspects of HMW subunit structure, such as lengths of domains and numbers and distributions of cross‐links. However, this requires a multidisciplinary approach bringing together molecular biologists, biochemists, and biophysicists to produce modified materials and determine their properties at the molecular level.

Higher level properties, determined by the interactions of polymers and monomers to give “aggregates/macropolymers,” are similarly challenging to explore but the use of methods to separate large molecules, such as asymmetric flow‐field flow fractionation together with varying the separation conditions to disrupt noncovalent interactions (Morel at al., 2020) should provide information on the relationships between composition, properties, and stabilizing forces.

These approaches may also be used to determine the effects of the environment on polymer assembly and properties and how these interact with genetic variation in gluten protein composition (Branlard et al., 2020).

However, it is likely that future improvements in quality will be incremental rather than “quantum leaps”: this is because the structure and properties of gluten have already been improved massively by a century of research applied to plant breeding.

## 4 PUROINDOLINES AND GRAIN TEXTURE

### 4.1 What do we know?

Grain texture (hardness) and protein content and quality are the two major traits that determine the end‐use quality of wheat grain and in particular the suitability for breadmaking. The importance of protein quality is readily understood and is discussed above. The importance of texture is less obvious to the nonexpert and relates to the relationships between grain structure, starch damage during milling, and the water absorption (WA) of flour.

The amount of water absorbed by flour (WA) is a key property exploited by bakers to optimize the mixing conditions. Bakers, therefore, specify the level of WA that they require for their flours and millers adjust their mills to achieve this level. WA is determined by the amounts of water absorbed by the individual grain components, with starch, protein, and fiber having the greatest effects. Starch damage occurs during milling and the extent of this is greater with hard wheat because they respond to higher grinding pressures during roller milling. This affects the absorption of water by starch, which absorbs three to four times its own weight of water when damaged compared with 0.5 times for undamaged starch.

Several breakthroughs contributed to the identification of the puroindoline proteins (Pins) as the major determinants of grain hardness.

**Figure 2:**
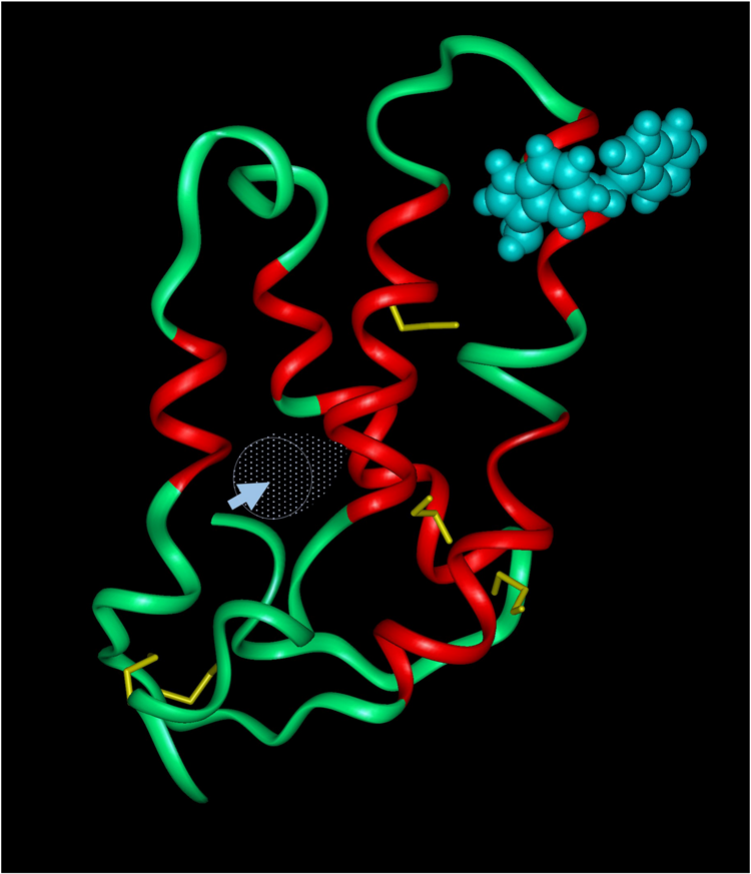
Possible molecular structure for wheat Pin b. The backbone of the protein is shown as a ribbon with alpha‐helical sections in red. The five disulfide bonds are shown as yellow sticks and the side chains of the tryptophan residues present in the “Lys.Trp.Trp.Lys” putative sugar binding motif are shown as blue overlapped van der Waals atoms. This structure also contains an internal hydrophobic cavity that may be involved in lipid‐binding. The relative position of this cavity is illustrated with blue dots with the entrance indicated by the blue circle and arrow. The structure was generated by homology modeling using a high‐resolution X‐ray crystal structure of a wheat nonspecific lipid transfer protein (ns‐LTP1; PDB accession: 1BWO) as a template. Hydrogen atoms were added consistent with pH 7 and side chain clashes were avoided by choosing low‐energy rotamers. The model was surrounded by a 5‐Å layer of water molecules, and the energy of the structure was minimized for 1000 cycles of conjugate gradient minimization using the CHARMM forcefield and CHARMm v.29b1 (Accelrys Inc.). The stereochemical quality of the model was assessed with the Biotech validation suite for protein structures and Procheck v.3.5 (Laskowski et al., 1993). The structure was generated by Dr. Frederic Beaudoin (Rothamsted Research) and is reproduced with his permission. [Color figure can be viewed at wileyonlinelibrary.com]]

The initial breakthrough was when Greenwell and Schofield (1986) compared protein fractions prepared by washing the surfaces of starch granules from hard and soft types of wheat. Although the granules were prepared using an aqueous procedure, they differed in the amount and composition of proteins bound to their surfaces, with a group of proteins with molecular weight by SDS‐PAGE of about 15,000 being present on the surfaces of the starch granules from soft but not hard wheat. These proteins were named “friabilin” to reflect their association with grain friability. Further analyses showed that the friabilin band separated by SDS‐PAGE comprises several components, with the second breakthrough being the demonstration that the most abundant of these components corresponded to two proteins that had been purified using a procedure designed to isolate proteins that were tightly bound to membrane lipids (Blochet et al., 1993). These two proteins each comprise about 120 amino acids, including six conserved cysteine residues, with molecular weights of about 13,000. They were called puroindolines (Pins) a and b to reflect their high contents of the amino acid tryptophan, with three (in Pin b) or five (in Pin a) tryptophan residues clustered in the sequences Trp.Pro.Thr.Lys.Trp.Trp.Lys and Trp.Arg.Trp.Trp.Lys.Trp.Trp.Lys, respectively. We do not know the three‐dimensional structures of the Pins, but they form part of the prolamin superfamily of plant proteins and we have detailed structures of related proteins such as the 0.19 ATI and nonspecific lipid transfer proteins (nsLTPs) (Douliez et al., 2000). Aligning the sequences of Pins and LTPs shows that the tryptophan‐rich sequences form a short insertion in the Pins and it has been suggested that they form a “tryptophan‐loop” exposed on the surface of the protein (as shown in the model structure in Figure 2). However, it should be emphasized that such models are not “true structures” but are constructed to allow the formulation and testing of hypotheses (in this case the sites of binding to starch and lipids).

The third breakthrough was the demonstration by Craig Morris and colleagues that variation in grain texture was related to mutations affecting the expression or sequences of the Pin proteins. Grain softness results from the presence of the wild‐type alleles of the genes and the most widespread mutations which result in hardness are the deletion of the Pin a gene and a single amino acid substitution (the replacement of glycine at position 46 with serine) in the Pin b protein. Many other mutations have since been reported which vary greatly in their frequency in wheat genotypes and may result in a range of subtle variation in grain hardness (reviewed by Morris et al., 2021; Morris, 2002).

### 4.2 What do we need to know?

These three advances are now generally accepted with variation in Pins estimated to account for about three‐quarters of the variation in wheat grain texture. By contrast, our understanding of the mechanistic aspects of Pins and hardness remains far from complete: this is despite a large volume of research, with some 175 publications with puroindoline in their titles listed on the Web of Knowledge between 1993 and 2022.

In particular, we still do not understand how the structures and interactions of Pins with other grain components result in the differences in the adhesion between the starch granule surface and protein matrix which occur between hard and soft types of wheat.

Three topics that may increase our understanding are briefly discussed below.

#### 4.2.1 What are the roles of lipids and the tryptophan loop?

It has been known for almost 30 years that hard and soft types of wheat differ in their contents of free polar lipids in flour (Panozzo et al., 1993) and a locus controlling free polar lipid content (*Fpl‐1*) appears to be closely linked or identical to the hardness (*Ha*) locus that encodes the Pins and controls hardness (Morrison et al., 1989). More recent analyses showed that the amounts of polar lipids bound to the surface of starch granules were greater when the wild‐type alleles of Pin a and Pin b were present and dramatically reduced when Pin b mutant or Pin a or Pin b null alleles were present (Finney et al., 2010). Pins were initially purified using a protocol developed for proteins associated with lipids (Blochet et al., 1993) and have been shown to bind phospholipids in vitro (Dubreil et al., 1997; Wilde et al., 1993; Clifton et al., 2007, 2008). It has also been shown that the binding in vitro involves the tryptophan loop (Clifton et al., 2007).

This has led to the assumption that the binding of lipid by the Pins, and specifically by the tryptophan loop, also occurs *in planta* contributing to the hardness mechanism. However, there is no direct evidence for this and it is perhaps more likely that the tryptophan loop is involved in starch binding as tryptophan residues are frequently present in starch‐binding domains of proteins (Janecek et al., 2019). The direct binding of the tryptophan‐rich regions of Pins to the starch granule surface has also been demonstrated by “tryptic shaving,” a procedure in which starch granules were treated with trypsin to digest exposed proteins and the protected (i.e., bound) fragments identified by mass spectrometry (Wall et al., 2010).

This does not, however, explain the role of lipids in hardness. It is notable that the amino acid sequences of Pins are related to those of the nonspecific lipid‐binding proteins (nsLTPs), a well‐characterized group of proteins present in seeds and other plant tissues. The three‐dimensional structures of nsLTPs comprise a bundle of four α‐helices with a large internal cavity that is capable of binding lipids and other hydrophobic molecules (Marion et al., 2005). We do not have three‐dimensional structures of Pins so it is not possible to conclude whether they bind lipids in vivo by a similar mechanism. However, the hypothetical structure shown in Figure 2, which was constructed using an LTP structure as a template, predicts the presence of a lipid‐binding pocket (see arrow) in Pins. If so, the relationship between polar lipid content and hardness may result from differences in the abundances of Pin proteins (and hence bound lipids) rather than a direct functional role of lipids.

#### 4.2.2 How do pins interact with gluten proteins?

The difference in the amount of energy required to mill hard and soft wheat relates to the degree of adhesion between the surfaces of the starch granules and the matrix of proteins in which these are embedded: this adhesion is stronger in hard wheat and more energy is required to release the granules, increasing the degree of granule damage (and hence WA). In vitro studies with purified Pin a showed that it formed large aggregates with gliadins and the authors suggested that “the interaction of Pin a with a monomeric gliadin creates a nucleation point leading to the aggregation of other gliadins, a phenomenon that could prevent further interaction of the storage prolamins with starch granule” (Geneix et al., 2020). The authors also showed that Pin a interacted in vitro with a recombinant pentapeptide based on a gliadin repeat motif (eight repeats of ProGlnGlnProTyr) and referred to reports that tryptophan residues may be involved in the interactions of proteins with proline‐rich domains (Ball et al., 2005). This article, therefore, suggests a third role for the tryptophan loop of Pins in binding gluten proteins. However, this role has not been established.

#### 4.2.3 How is “hardness” established during grain development

The Pin story illustrates the challenges in studying complex multicomponent biological systems and these challenges are exacerbated by the nature of the dry mature grain. It is possible, however, to increase our understanding by focusing on grain development, during which the initial interactions may be established.

Pins are synthesized with N‐terminal signal peptides which direct the newly synthesized protein into the lumen of the endoplasmic reticulum (and are cleaved as part of the process). They are, therefore “secretory proteins” and would be expected to be transported into the storage vacuoles where interactions with gliadin proteins could be initiated. Their interactions with gliadins could therefore be established in the growing protein bodies, as suggested by Geneix et al. (2020). Their movement to the surface of the starch granules presumably occurs during grain desiccation, when the cells of the starchy endosperm die and their contents become disrupted. During this phase, the water content of the grain falls to 15% or below but there is initially sufficient water to allow diffusion of the water‐soluble Pins (and perhaps small Pin:gliadin aggregates) to the starch granule surface. Interactions with lipids, if they occur at all *in planta*, may take place during the same phase, perhaps with the polar lipids derived from the double membranes of the amyloplasts in which the starch granules are synthesized. Hence, it is important to understand the cell biology of the developing grain to explain the functional properties at maturity.

### 4.3 How can we answer these questions?

The molecular basis for grain texture is one of the most fascinating questions in cereal chemistry and one of the most difficult to answer due to the multiple components and the challenges of exploring interactions in the “natural” state. Analyses of purified Pins have failed to give definitive information on their molecular interactions *in planta*, showing that the tryptophan loop interacts with lipids which contrasts with a role in starch binding which has been demonstrated by tryptic shaving. However, even the latter results are not definitive as the Pins are soluble in aqueous media and their distribution may therefore be affected by the water‐washing that is used to prepare starch granules. It is therefore important that studies of the interactions of Pins with starch granules should be confirmed by analyses of granules prepared without washing (Darlington et al., 2020).

Assuming that the tryptophan loop is responsible for the binding of Pins to starch granules, it is possible to propose models for the interactions of the four components of the system: starch granules, Pins, gluten proteins, and polar lipids. These interactions could be explored by combining imaging of developing and mature grain with analyses of native (i.e., unwashed) starch granules, using transgenesis or gene editing to generate plants expressing mutant forms of Pins. However, such studies are expensive and difficult, being at the limit of current analytical and imaging systems.

Bearing these financial and practical considerations in mind it is perhaps doubtful whether the mechanism will ever be fully understood. This is because the interest in Pins is largely academic rather than driven by applications, as a range of variations in texture is currently available to plant breeders and there is commercial interest in identifying new properties.

## 5 ADVERSE REACTIONS TO WHEAT PROTEINS

The most dramatic development in wheat research over the past few decades has been the increased emphasis on adverse reactions to wheat consumption. This has focused particularly on two groups of proteins: gluten proteins and ATIs (discussed below).

### 5.1 Gluten proteins

Classical IgE‐mediated allergy to wheat is comparatively rare, probably below 0.25% (Zuidmeer et al., 2008), and allergy tends to occur in children and then be outgrown. Over 30 individual wheat proteins have been identified as causing IgE‐related sensitization to wheat‐based foods including gluten proteins (Gilissen et al., 2014; Tatham & Shewry, 2008). Although the symptoms of allergic reactions to wheat are generally mild, one rare form results in a severe reaction. This is wheat‐dependent exercise‐induced anaphylaxis (WDEIA). The major allergens in WDEIA are ω5‐gliadins and HMW‐GS, but other gluten proteins may also sensitize some patients (reviewed by Scherf et al., 2016). Hence, although it is possible to silence ω5‐gliadin (Altenbach et al., 2014) and HMW‐GS genes (Lafiandra & Shewry, 2022) this approach is unlikely to be used to develop hypoallergenic wheat.

By contrast, there is currently significant interest in producing wheat suitable for those with celiac disease (CD). CD is a well‐characterized condition that was first described in ancient Greece. The association with wheat was first described in the 1940s and with wheat gluten in 1952. It affects about 1% of the global population and is a T‐cell‐mediated autoimmune response that is triggered by short (nine amino acid) peptide sequences (epitopes) present in gluten proteins. Almost 40 such sequences have been identified in wheat and related cereals (Sollid et al., 2020). They occur in all types of gluten proteins, although the numbers of epitopes vary between and within different gluten protein types (Shewry & Tatham, 2016). The contents of celiac‐active gliadins can be reduced by RNAi‐mediated suppression (Gil‐Humanes et al., 2010) while CRISPR/Cas9 gene editing technology has been used to target celiac epitopes within gluten proteins (Sanchez‐Leon et al., 2018). However, the complexity of the gluten protein multigene families means that substantial modifications will be required to produce “celiac‐safe” wheat.

The use of modern molecular breeding methods to reduce or eliminate celiac epitopes from wheat gluten proteins would almost certainly have an impact on grain processing quality and on the cost of the modified wheat. The development of such modified lines also presents a challenge for wheat breeders to incorporate complex traits in their breeding programs. Consequently, the most likely outcome will be the development of “celiac‐safe” wheat as a high‐cost niche product which will limit its availability to celiac patients. Hence, the avoidance of wheat and gluten and the development of foods from gluten‐free crops are likely to continue to be the major strategies for celiac patients in the medium term.

### 5.2 ATIs

The ATIs are the most abundant group of water‐soluble (albumin) proteins in wheat, accounting for 2%–4% of the total grain proteins (Dupont et al., 2011). They are considered to provide a broad spectrum but low‐level resistance to pests and pathogens that threaten the developing and mature grains, together with other proteins and nonprotein components (Shewry & Lucas, 1997).

The ATIs were first described in 1943 and a large volume of literature has since provided details of their polymorphism, genetics, and properties. This has been reviewed in detail by Geisslitz et al. (2021) and can be briefly summarised as follows.

The ATIs are small proteins (with masses ranging from 10,000 to 16,000) and are present in the grain as monomers and in dimeric and tetrameric forms. Nine forms have been defined based on differences in their amino acid sequences and activities but at least some of these occur in multiple isoforms and at least one form may be glycosylated. Hence the total number of ATIs present in single genotypes has been reported to range up to 19 (Altenbach et al., 2011). They include monomeric subunits that inhibit only trypsin (TIs) but most inhibit both proteases and α‐amylases from mammals (including humans) and insects (Coleoptera and Lepidoptera) but not endogenous α‐amylases of wheat. Their nomenclature is confusing for the nonexpert, with the subunits present as monomers and dimers being called 0.19, 0.28, and 0.53 based on their electrophoretic mobilities and the subunits present as tetramers called CM proteins because they were initially identified as soluble in a mixture of chloroform and methanol. A brief summary is presented in Table 1.

**Table 1:** Nomenclature and properties of wheat ATIs

Aggregation states		Protein subunits (forms)	Widely used name	Allergenic activity (at least one form)		% total flour protein	Amylase inhibitory activity
				Bakers' asthma	Food allergy		
Monomeric		WMAI‐1	0.28	Yes	Yes	0.5	Insect (Coleoptera and Lepidoptera) amylases and human salivary and pancreatic amylases but vary in relative activity
Homodimeric		WDAI‐1	0.53	No	Yes	1.0	
		WDAI‐2	0.19	Yes	Yes		
Tetrameric	1st subunit	WTAI‐CM1	CM1	Yes	Yes	1.7	
		WTAI‐CM2	CM2	Yes	Yes		
	2nd subunit	WTAI‐CM16^a^	CM16	Yes	Yes		
		WTAI‐CM17	CM17	Yes	Yes		
	3rd subunit (2 copies)	WTAI‐CM3	CM3	Yes	Yes		
Monomeric		CMX1/2/3	TI	No	Yes	0.2	none

*Note*: Some subunits (forms) exist in multiple isoforms, which may differ in their ability to induce allergic responses to food and on inhalation. Hence, “allergenic activity” is based on the demonstrated activity for at least one isoform. Based on Tables and sources reported by Geisslitz et al (2021).

^a^ CM16 occurs in glycosylated and nonglycosylated forms.

Interest in ATIs has increased dramatically over the last decade and they are currently the most active topic in cereal research. This is because of their role in triggering adverse responses in humans, including the three major types: allergy, celiac disease (CD), and noncoeliac wheat sensitivity (NCWS).

The most widespread allergic response to wheat is not food allergy (as discussed above under gluten proteins) but an allergic airways response to inhalation of flour and dust which is generally referred to as bakers' asthma. This is a major occupational disease among workers who handle wheat grain, particularly those working in ill‐ventilated mills. For example, bakers' asthma is the second most common type of occupational asthma in the United Kingdom. At least seven forms of ATIs, including monomeric, dimeric, and tetrameric types and TIs, have been reported to trigger bakers' asthma with glycosylated forms being particularly active (Table 1). Similarly, all major forms of ATI have been shown to cause IgE‐related sensitization to foods (Geisslitz et al.,^2021^) (Table 1).

CD is a well‐defined condition and there is no doubt that the major triggers are glutamine‐rich sequences in gluten proteins (Sollid et al., 2020) (as discussed above). However, it has been suggested that ATIs also play a role in eliciting an innate immune response (Junker et al., 2012).

In contrast to allergy and CD, wheat sensitivity is a poorly defined condition with a range of reported symptoms (including gastrointestinal symptoms, tiredness, headache, dermatitis, pains in muscles and joints, depression, anxiety, and anemia) (Sapone et al., 2012). The diagnosis of NCWS is therefore difficult and most patients are self‐diagnosed. Hence, the prevalence is difficult to determine and estimates vary widely (from about 1% to 10% of the population) (Brouns et al., 2019).

The etiology of NCGS/NCWS is not understood but is likely to feature a mixture of factors including the stimulation of the innate immune system. A range of causative substances have been suggested, including gluten proteins and fermentable sugars (Fermentable Oligosaccharides, Disaccharides, Monosaccharides, And PolyolS, often called FODMAPs). However, current attention is focused on the role of ATIs, which have been suggested to induce an innate immune response (Schuppan & Gisbert‐Schuppan, 2019).

The literature on NCWS, ATIs, and human health is increasing rapidly and a detailed discussion is outside the scope of this article. However, several points should be noted.

First, ATIs are difficult to prepare in sufficient amounts and levels of purity for use in experimental studies, particularly in humans, and most of our information is based on studies in animal models or the use of impure fractions. Similarly, ATIs may be present as contaminants in gluten fractions used for challenges.

Second, ATIs are only one of many types of wheat grain proteins which have biological activity against invading organisms. These include lectins, nsLTPs, ribosome‐inhibiting proteins (RIPs, which are related to castor bean ricin), thionins, defensins, proteins related to the sweet protein thaumatin, and several families of protease inhibitors, including the bifunctional wheat amylase‐subtilisin inhibitor (BASI) (see reviews by Brijs et al., 2009; Shewry & Lucas, 1997; Shewry et al., 2009). Although most of these proteins are present in low concentrations in the grain compared with ATIs and gluten proteins, they could be present as contaminants in ATI and gluten preparations used for the determination of biological activity.

Third, we know nothing about the molecular basis for the proposed biological activity of ATIs, in terms of structure:activity relationships.

### 5.3 Challenges to understanding the role of wheat proteins in adverse reactions to wheat

The three conditions discussed all appear to be immune‐mediated but affect different components of the immune system: immunoglobulin E (IgE)‐mediated allergy, T‐cell‐mediated autoimmunity, and (possibly) the innate immune system for NCWS.

Wheat gluten proteins are clearly implicated in two of these conditions, allergy and coeliac disease, and epitopes have been identified in the glutamine‐rich repetitive domains of the proteins (reviewed by Scherf et al., 2016). However, there is no evidence that the two conditions are triggered by the same epitopes.

ATIs have been implicated in triggering all three reactions but in this case, we know little about the mechanisms or the structural features of ATIs that determine their activity. For example, does the fact that ATIs but not the closely related TIs (as far as we know) trigger NCWS imply that the amylase‐inhibitory domain/site is a key feature? If this is the case it is important to determine whether other inhibitors of α‐amylase also exhibit activity.

The three‐dimensional structure of one ATI is available, the 0.19 subunit which occurs as a homodimer (Oda et al., 1997). However, the high level of sequence homology between ATI forms means that this can be used as a basis for developing structural models for other forms and exploring the structural basis for biological activity. The development of heterologous expression systems should also allow the production of pure proteins for experimental studies, including mutants to explore structure:function relationships. However, it is difficult to envisage how this understanding can be achieved without reliable in vitro test systems. Understanding structure:function relationships should also facilitate the development of safer wheat by using gene editing to make precise changes to the sequences and structures of ATIs, instead of aiming for complete eliminations as in recent studies using transgenesis (Kalunke et al., 2020) and gene editing (Camerlengo et al., 2020).

## 6 CONCLUSIONS

Despite over a century of research, there are still major limitations in our knowledge of the structure and functionality of wheat grain proteins and their role in diet and health. The major limitation for improving grain functional properties is understanding how the individual gliadin and glutenin proteins are assembled to form polymers and higher order aggregates/macropolymers, how the assembly is controlled by genetic and environmental factors, and how the structures formed determine dough properties. Understanding the impact of the environment is becoming increasingly important because of the greater frequency of extreme weather events associated with climate change. Hence, improving the stability of grain quality is a more important target for the future than improving intrinsic quality.

The major challenge for reducing adverse impacts on health is to determine how the ATIs and, to a lesser extent, gluten proteins trigger responses in susceptible individuals. This will allow us to design strategies to reduce adverse effects, using either plant breeding and biotechnology or innovative processing to remove or inactivate epitopes. This should reduce the decline in the consumption of wheat‐based foods that is occurring in many countries.
